# Dental professionals’ perception of their role in the practice of oral health promotion: a qualitative interview study

**DOI:** 10.1186/s12903-023-02756-y

**Published:** 2023-01-25

**Authors:** Elena Shmarina, Dan Ericson, Bengt Götrick, Cecilia Franzén

**Affiliations:** 1 Department of Oral Diagnostics, Faculty of Odontology, Malmö University, Malmö, Sweden; 2 Kalmar County Council, Public Dental Service, Oskarshamn, Sweden; 3 Department of Cariology, Faculty of Odontology, Malmö University, Malmö, Sweden

**Keywords:** Salutogenesis, Ottawa charter, Health promotion

## Abstract

**Objective:**

To explore dental professionals’ perceptions of their role in the practice of oral health promotion.

**Material and method:**

In-depth interviews were conducted with three dentists, one specialist dentist and seven dental hygienists. All were employed in the public dental service in Kalmar County, Sweden and had at least 2 years’ work experience. The interview questions addressed the experience and views of dental professionals with reference to their role in the practice of health promotion. The interview data were subjected to qualitative content analysis.

**Results:**

Analysis revealed two themes which capture the essence of the dental professionals’ perception of their role in the practice of oral health promotion. One theme, *having person-focused approach*, comprised four categories: ‘considering the patient’s life situation’, ‘establishing a trusting relationship with patients’, ‘strengthening patients’ commitment to oral health’ and ‘health education’. The other theme, *perceiving social responsibility for oral health,* comprised three categories: ‘dissemination of oral health knowledge’, ‘interprofessional collaboration’ and ‘equality in oral health care’*.*

**Conclusion:**

Dental professionals perceived promotion of oral health to be an important aspect of their professional role. They aspired to patient participation in the decision-making process and educational activities, as well as practising and evaluating skills development. Although the dental professionals perceived that they undertook health promotion activities, they did not clearly distinguish between oral health promotion and disease prevention. There was intra- and inter-professional agreement among the dentists and dental hygienists with respect to expected outcomes for health promotion activities.

## Introduction

World Health Organization (WHO) has acknowledged the unique role of the research community [1] and oral health professionals [2] in promoting oral health. However, little is known of the dental professionals’ perception of their health promotion practice, despite their important role in oral health promotion at an individual level: this in turn influences the oral health of the population. Moreover, our previous study suggests that elderly dentate individuals with good oral health recognise the important role of dental professionals in promoting oral health throughout their lives [3]. Thus, it is important to gain more understanding of how dental professionals perceive their health promotion role in the context of daily dental practice. This understanding can enhance discussion, not only about values and attitudes which influence the current dental care system, but also about further development of oral health promotion activities.

Health promotion is defined as the process of enabling people to increase control over, and to improve their health [4]. The Ottawa Charter, a central document of health promotion, outlined the philosophy and guiding principles for health promotion. Initially however, there was no clear theoretical framework supporting these principles [5]. Subsequently, the development of health promotion was substantially influenced by Antonovsky’s salutogenic theory [6, 7], which focuses on resources for health and health-promoting processes. The philosophy underlying the salutogenic theory corresponded well with the essence of the Ottawa Charter [5].

To describe salutogenesis in the context of health promotion, Eriksson and Lindström (2008) developed a metaphor ‘Health in the river of life’ [5]. It comprised a number of stages, from *health promotion, health education, disease prevention, disease protection* to *treatment* and was an uncomplicated way of illustrating the shift in focus towards health. Our study was based primarily on *oral health promotion*, *oral health education* and *oral disease prevention*. The *prevention* stage aims at reducing the negative effects and risks of oral diseases, thus maintaining oral health. The interventions are both population-based, such as fluoride mouth-rinsing in schools, and individual-based, such as brushing with fluoride toothpaste. The *oral health education* stage is based on a dialogue, wherein people make their own health decisions, with the support of dental professionals. The main focus is on improving oral health literacy at both population and individual levels. In the *health promotion* stage, health education progresses to a continuous learning process in which dental professionals engage in a creative interplay with individuals and groups, in order to identify and mobilise resources which promote health and well-being [5, 8]. In chairside oral health counselling, the patient’s own resources and experiences should be given priority, rather than a focus solely on an oral diagnosis [9].

To date, research on oral health promotion has concerned primarily preventive interventions [10, 11], capacity development [12, 13] and settings and policy approaches [14]. There are few published studies exploring dental professionals’ perspectives on health promotion and they are quantitative studies [15, 16]. For example, a cross-sectional study focusing on oral health professionals’ views on health promotion showed that the majority of participants perceived oral health promotion to be part of their role, but they felt ill-equipped to apply it [15]. Another study showed most dental professionals regarded ‘oral health promotion’ and ‘oral health education’ as synonymous. The participants used clinical prevention and educational methods and reported spending less than two hours a week on these activities. It has therefore been proposed that a qualitative approach should be adopted to research in this field, in order to gain a deeper insight into dental professionals’ perceptions of their role in health promotion [16].

## Aim

To explore dental professionals’ perceptions of their role in the practice of oral health promotion.

## Material and method

We used the Consolidated Criteria for Reporting Qualitative research (COREQ) as a reporting guideline [17]. Building on an interpretive approach, we applied qualitative design using individual in-depth interviews [18]. The conceptual framework was based on guiding principles for health promotion outlined in the Ottawa Charter [4] and on the concept of salutogenic orientation as described by Antonovsky [7].

### Settings

The study was conducted in Kalmar County, Sweden. The county council administers two specialist and eighteen general public dental clinics, providing dental care for 247,000 inhabitants.

### Participants

Purposive sampling was used to identify potential participants. Purposive sampling was chosen because it made it possible to select most appropriate participants for the study who had experience of health promotion activities and could provide rich information on the matter [19].

The participants comprised registered dental professionals employed in the public dental service in Kalmar County, with at least 2 years’ work experience. The participants were recruited between September and December 2021 through managers’ and personal contacts at both the general and specialist public dental clinics. All approached participants approved.

A total of 11 registered dental care professionals (9 women and 2 men) participated in this study: three general dental practitioners, one specialist dentist and seven dental hygienists. Their work experience ranged from 3 to 34 years (median 12).

### Data collection

Individual in-depth interviews were conducted by the first author (dental hygienist) and addressed dental professionals’ experience and views of their role in health-promoting practice. A basic interview guide was developed in consultation with all the authors. The interview questions were: ‘How do you view your own role in promoting oral health for your patients?’ and ‘Do you think that there is a health-promoting approach in dental care?’ The participants were also encouraged to describe and reflect on situations relevant to promotion of oral health in their patients. The participants’ answers were followed up with clarifying and exploratory questions, such as, ‘Could you tell me more?’ or ‘How did it feel?’.

The interviews were conducted over a period of four months, from September to December 2021, during the Covid-19 pandemic, using Zoom and Skype life video meetings. Recruitment and interviewing were carried on consecutively until data saturation was reached. No further interviews were conducted once the newly collected data tended to repeat the data already collected [20]. All interviews were audio‐recorded and transcribed verbatim by the first author. The interviews lasted from 51 to 96 min (median 61).

### Data analysis

The transcribed data were organized and processed in NVIVO (Release 1.5.1) software, and analysed using an inductive approach to qualitative content analysis, as described by Graneheim and Lundman (2004) [18].

The analysis was conducted in several steps. Firstly, the transcripts were read through several times to obtain a sense of the whole and to identify the meaning units dealing with the dental professional’s role in health promotion. A meaning unit comprises several words, sentences or paragraphs related to each other through their content and context [18]. Next, each meaning unit was condensed in such a way that it did not lose its content and was then labelled with a code. The codes were compared for differences and similarities, and codes with similar content were then stratified into categories and subcategories. Thereafter, the underlying meaning, the latent content of the categories was read, critically analysed, and formulated into themes. A theme is a thread of meaning running through a category at an interpretative level [18]. Throughout the analysis process, the codes, categories and themes were initially identified by the first author and thereafter discussed by all authors until consensus was achieved.

## Findings

The interview data revealed two themes which capture the essence of the dental professionals’ perception of their role in the practice of oral health promotion: *having person-focused approach* and *perceiving social responsibility for oral health*. Table 1 summarizes the formulated themes and the associated categories which emerged from the analyses. Direct quotations from the interviews are included to illustrate how the interpretation was grounded in the data. Quotations are identified by the abbreviation D for dentist and DH for dental hygienist and a number assigned to each participant. The quotations were translated from Swedish into English by a bilingual, dentally qualified professional translator.

**Table 1:** Overview themes and categories

Theme	Category
*Having person-focused approach*	Considering the patient’s life situation Establishing a trusting relationship with the patient Strengthening patient’s commitment to oral health Health education
*Perceiving social responsibility for oral health*	Dissemination of oral health knowledge Interprofessional collaboration Equal oral health care

### Having person-focused approach

This theme describes the principles highlighted by the participants as important for promoting oral health in their patients. There are four categories: *considering the patient’s life situation*, *establishing a trusting relationship with the patient*, *strengthening the patient’s commitment to oral health* and *health education*.

#### Considering the patient’s life situation

The data revealed an understanding and awareness that taking the patient’s life situation into account is important for achieving good oral health. The participants said that personal information is needed to understand the patient’s life situation and talked of coming to an understanding of the patient’s situation in order to plan and implement appropriate health promotion activities.

Listen to the patient describing where they are at in life because patients have different routine, different jobs. Yes, there is a huge difference from patient to patient. What one as a professional can demand of a patient and what there is to build on (DH5)

#### Establishing a trusting relationship with the patient

The participants emphasised the importance of establishing a trusting relationship with patients. They perceived it to be a key element in the health-promoting process and stated that it helps to recognize and adapt to the patient’s needs and influences the outcomes of health promotion initiatives.

This contact is important. It is hugely important to establish trust. If you can establish trust you will find out a great deal and then you can adjust your approach to the situation in which the patient finds him- or herself (DH5)

#### Strengthening patients’ commitment to oral health

As part of oral health promotion work, the participants described different intertwined approaches which they applied to strengthen the patients’ involvement in his or her own oral health. They talked about cooperation, coaching, supporting, following up. Prioritizing communication with a patient was mentioned as a key instrument in health promotion. There was frequent emphasis on dialogue with the patient as the most important aspect of chair-side counselling. According to one dental hygienist:

Everyone says you just need to splash a bit of fluoride around, but for me that is the last thing I do before the patient leaves the surgery. Of course we haven’t sat in silence for the previous 30 minutes, the entire consultation with the patient is about following up and giving guidance and establishing a common goal and achieving an understanding of what we are about (DH10)

The participants spoke about the importance of working in cooperation with patients and treating them with respect. They perceived that to focus solely on clinical measures would not be effective in the long term. What mattered was to make person to person contact, to be compassionate, motivating and work together with the patient towards common oral health goals. The participants described their health-promoting approach and perception of being an important part of it, such as this dental hygienist:

Explain so that they understand, don’t be accusatory, but reach out to them and then you can become a team together with the patient, striving for the same goal. That is what to aim for and then all patients have of course different goals. But I think we have a very big role to play here. In any case that’s how I feel in relation to my patients (DH2)

There was frequent emphasis on facilitating patient involvement as an important part of health promoting activities. The participants described gaining patients’ interest and understanding, which, they perceived, would make the patients feel involved in their own oral health care.

Some participants perceived it in terms of health gain, such as this dental hygienist:

If you can reach them here and they understand their own role in this context and you get through to them and you can support and assist, that’s the prize (DH3)

These participants described their health promotion roles differently, depending on their profession. Several participants talked about the importance of spending more time with patients to encourage active patient participation. They perceived that they would succeed in promoting oral health if they put time and effort into fully focussing on the patient. The participants emphasized the importance of being patient with patients. They talked about not giving up in the absence of cooperation, but to keep on developing rapport with patients, to support them and enable them to maintain their oral health.

Then you’ll probably have to struggle on for a while and maybe you won’t get anywhere the first time, or the second or the third, but the fourth time, then the patient starts to understand a little. Sometimes you have to be a bit persistent and stubborn and not give up (D8)

Besides that, support was often mentioned as an essential part of their health promoting work. Some talked about confirming patients’ effort and always finding something positive to build on, while others described it as a springboard for patients, such as this dental hygienist:

I am just a sort of springboard or perhaps I should say a motivator for them. Sometimes I just open their eyes or something. I usually say that I am their biggest supporter. How can I describe it, I stand on the sidelines and of course I applaud what they do. I always give patients the opportunity to decide for themselves (DH10)

#### Health education

The participants viewed the oral health education that they offered to their patients as closely linked to the patient’s oral health status. They talked about providing their patients with opportunities to acquire oral health information and skills which are conducive to good oral health, as well as about promoting health benefits and helping patients to adopt an attitude of active self-care. A dentist with long work experience said:

My duty is really to try to get them to understand the consequences of our behaviour. What it can lead to in the long term (D7)

Many participants emphasised the importance of providing information to all kinds of patients, regardless of their state of health, both orally healthy and diseased. A dental hygienist stated that operative treatments have no major effect on either caries or periodontitis if no information is provided to the patient. She said:

Treatment without information that has no great effect on either caries or periodontitis. The information is in fact so important and so is reaching out to everyone (DH2)

Another pointed out that to maintain oral health, even orally healthy patients should be informed about potential health risks. She said:

It can in fact be very important that we chat a little with the healthy patients we see, that we still inform them about what is healthy, so that they don’t start acquiring unhealthy habits like drinking soft drinks and so on (DH4)

All participants talked about the importance of customizing oral health information and using a pedagogical approach in oral health education. One dental hygienist spoke about providing information over several appointments, as she believed that no one could assimilate all the information at the one appointment. Another talked about the importance of knowing the patient, in order to customize the information in the most suitable way. She said:

Giving information is really like putting together a packet which that particular patient needs in order to take it in and with some, as I said a little earlier, you have to be tough…now this is the way it is, like and with some patients you have to wrap it up in silk so that they are able to take it in and with some you have to do a little at a time. Others want the full story straight off (DH6)

Describing their pedagogical approach, the participants talked about complementing the verbal message with other ways of delivering patient education, such as demonstrations, x-ray images or printed materials. A dental hygienist commented that words alone might disappear and using images or printed materials might make it easier for patients to understand the oral message. Another talked about the importance of individual interaction with a patient and tailoring instructions to the patient’s personal pace and learning needs. She said:

I try to express it another way, use the mirror a lot: “This is what it looks like, and what has caused it”, and yes, I try to explain in another way and dare to put open questions. ”What do you believe yourself is the reason you get so many holes?” and then you have to follow-up on this and not let it go (DH 4)

As a part of health promotion activity, the participants described how they worked on developing their patients’ personal skills, helping them find motivation and customizing oral hygiene self-care recommendations. They talked about trying different ways of instructing and trying out various cleaning devices to identify those which would be best suited to the particular patient.

But then in fact you have to try to find something which they really can manage to use at home and make it part of their self-care routine at home, so that one can make it easier in some way, for example help them to find a good toothbrush or help them establish some sort of routine. Because it is really hard. If you add up all the time they put in at home, then that is what matters most. Its a matter of finding the right aids and the motivation (DH9)

### Perceiving social responsibility for oral health

This theme covers the participants’ perception of their responsibility for oral health at population level, as part of a dental care system. The theme expressed itself in focusing on promotion of oral health at different levels of society, pursuing equality in oral health and interprofessional collaboration. This theme has the following three categories: *dissemination of oral health knowledge*, *delivering equal oral health care* and *interprofessional collaboration.*

#### Dissemination of oral health knowledge

The participants talked about using various channels to spread oral health information to target audiences. One dental hygienist described lecturing on oral health to various groups of people, such as patient associations or student nurses as part of her health promotion work. She said:

Others also need to understand, other healthcare professionals must also understand the associations that exist (DH3)

Another described visiting schools and preschools, to talk about oral health to both children and their parents. There was emphasis on the importance of reaching all populations and all ages to pursue good oral health in the population.

We also work at the population level. And I think that is also incredibly important, that we cover the full spectrum. Meaning that we are everywhere and we become a natural part of it (DH2)

All participants had positive attitudes to health promotion and perceived oral health promotion to be an important aspect of their professional role, such as this dentist:

If we don’t look after those who are healthy, then there will be more sick people (D11)

However, several participants talked about the differences between dentists’ and dental hygienists’ education with respect to health promotional programs, as well as differences in education in years gone by. One dentist with many years of work experience expressed the hope that newly graduated dentists would be more suited to health promotion work. Another, describing her education, said:

There was in fact not a lot about health promotion. No, there wasn’t, in fact. There was much greater focus the technical side, on treatment, preps and things like that (D11)

One dentist commented that dental hygienists’ training had a greater focus on health promotion activities than the education of dentists. Another observed changes in dental hygienist education over time, with greater emphasis on health promotion:

I see that the hygienists who are graduating now, have, in fact, I think they have a completely different attitude to the health aspects (D7)

#### Interprofessional collaboration

The participants emphasized interprofessional collaboration as an essential part of oral health promotion work. They spoke about collaboration on different levels, not only within the dental health sector, but also with the health sector. Some participants talked about collaboration among different dental professions with the patient at the centre of care. Other participants described collaboration with professionals from other sectors.

I think it’s important to try to collaborate a bit more over the professional boundaries and when it comes to children’s dentistry I think it’s really very important to work together with the schools and Child Health and so on, and with the elderly. That dental care is promoted a little more (D7)

I would like to see us go hand in hand more with domiciliary health services, home assistance and elderly care (DH9)

#### Equal oral health care

The participants often mentioned the importance of pursuing equality in oral health: that every patient should have the opportunity to be as healthy as possible and they saw their role as being inclusive, such as this dentist:

I don’t differentiate between individuals at all. I really try to treat all individuals according to the same treatment principles (D8)

However, the participants perceived that some barriers to implementation of health promotion in dental practice, are beyond their power, for example allocation of resources or shortage of dental staff limit the pursuit of equality in oral health. Several participants expressed concerns about not being able to offer equal oral health care. In their opinion, the solutions to these problems were in the hands of employers:

We can’t fix that, us workers. That’s what the employers have to do that and this issue of resources in in fact a very big issue (D1)

## Discussion

This study explored dental professionals’ perceptions of their role in the practice of oral health promotion. Overall, the findings indicate that dental professionals are committed to oral health promotion. This commitment is expressed in recognizing oral health and its maintenance as a major social investment and a challenge, as well as in focusing on promotion of oral health in different levels of society and achieving equality in oral health. Two themes emerged in the analysis, *having person-focused approach,* and *perceiving social responsibility for oral health*, which indicated adherence to the practice of oral health promotion on two levels, individual and societal.

To achieve optimal oral health, individuals and their social well-being must be at the centre of decision-making, including understanding influential factors outside the clinical setting [21, 22]. Traditionally, patient oral health goals often comprise behavioural changes formulated solely by dental professionals [23]. However, the findings in this study demonstrate that the dental professionals in our sample strive to help patients identify their own goals and resources in order to maintain good oral health. They emphasised the importance of considering the patient’s life situation in orderto create a trustful relationship, which is beneficial in strengthening patients’ engagement in their own oral health. It has previously been documented that improving the listening and communication skills of health care professionals might boost patients’ self-efficacy, resolve feelings of resignation, and improve manageability [24].

The findings indicated that the participants regarded oral health promotion as an important aspect of their professional role. There was an understanding about the activities dental professionals should undertake in their role in health promotion practice. According to the literature this is an important prerequisite for health promotion [25]. At societal level, dental professionals engaged in community-based work, developing social environments and interprofessional collaboration to support oral health in the wider population. These health promotion activities are in accordance with the proposals in the Swedish national guidelines and in the Ottawa Charter [4]. At individual level, the participants perceived that education they offered their patients is closely linked to the patient’s level of oral health. The participants provided a number of detailed activity examples of how they work with developing personal skills by intertwining oral health education with coaching, following up, supporting and motivating. Effective application of these strategies requires relevant competencies. Kay et al. suggested that greater emphasis on teaching dental professionals about health psychology would make oral health promotion more effective [26]. Many Swedish dental professionals have the competence needed to undertake health promotion as they are trained in supporting behavioural changes, such as counselling or therapeutic approaches, and can offer support for changing unhealthy habits and promoting healthier behaviour. However, the participants described their health promotion role differently, depending on profession. Compared to the dentists, the dental hygienists in this study spoke more about health promotion as part of their daily practice. This difference might be due to differences in professional competencies and work tasks, where the dentist’s focus is on operative treatment, while the dental hygienist’s is on health promotion and prevention. However, comparing different professional groups was beyond the scope of this study and requires further research.

In addition, the findings indicate that the participants do not clearly distinguish between oral health promotion and disease prevention, but use these terms in conjunction when describing their health promoting activities. This aspect of findings reflects the use of these terms in the Swedish national guidelines for dental care [9]. However, it has been suggested that different interpretations of what constitutes health promotion can lead to misunderstandings and pose barriers to further development of the practice of health promotion [27].

With respect to implementation of health promotion in dental practice, the participants perceived some barriers which are beyond their control, such as allocation of resources or shortage of dental staff. The solutions are in the hands of employers, policy makers or governments. The participants would like resource allocation by dental care organisations to respond more proactively, to develop a broader range of health promotion practice activities as well as allocating more resources to ongoing activities. It has previously been reported that shortage of dentists is a major barrier to capacity building in public health and the organization of population-based programs for oral disease prevention and health promotion [28–30]. Our participants however, perceived that they carry out the health promotion activities that are within their power, such as considering the patient’s situation, identifying salutogenic factors and mobilising the patient’s’ own resources.

One limitation of this study could be the use of purposive sampling for recruitment: those who chose to participate might be more committed to health promotion than the average dental professional. Therefore, our sample may not be representative of either group of dental professionals. On the other hand, choosing participants with diverse experience improves the potential to shed light on the research question from a variety of aspects [18, 31]. Our participants were similar in that they all worked in the public dental sector and were employed in the same region. However, the participants’ different professions, different working life experience and different ages and sex, contributed to a richer variation of the phenomena. The study was undertaken in a Swedish context, which could influence the participants’ perception of their role in health promotion. The perception of our participants might differ from that of other dental professionals in a different context. All interviews and transcriptions were undertaken by the first author. This could influence the interview conditions and the analysis. However, the interviews, the coding and the categorization were discussed by the authors until consensus was achieved. Our interpretation of the interview data should be considered as one possible interpretation, as a text never implies one single meaning, just the most probable meaning from a particular perspective [32]. Quotations from the interviews have also been included to help readers make their own judgment. All the interviews followed the interview guide. They were also conducted and transcribed in a relatively short period of time: these factors reduce the risk of inconsistency of data collection [18]. The interviewer and the participants worked in the same organisation. Most of the participants were therefore already known to the interviewer and this might pose a potential risk for bias [33]. However, contact was limited to occasional meetings for educational purposes and referral of patients. None of the participants has ever worked at the same clinic as the interviewer and there were no established personal relationships.

## Conclusion

The findings indicated that dental professionals perceived oral health promotion to be an important aspect of their professional role. They recognized that oral health and its maintenance are a major social investment and challenge. They commented on their commitment to promotion of oral health at different levels of society and to achieving equality in oral health.

There was an understanding as to what activities dental professionals should engage in to accomplish their role in the practice of chair-side oral health promotion. The focus was on patient participation in the decision-making process and educational activities, as well as on practising and evaluating skills development.

Although dental professionals perceived that they carried out health promotion activities, they did not clearly distinguish between the terms ‘oral health promotion’ and ‘disease prevention’. Yet, using the same terminology may facilitate communication among all actors, from patients to national knowledge management. In addition, there was intra- and inter-professional agreement among dentists and dental hygienists with respect to expectations for health promotion role activities.

## Data Availability

The datasets used and/or analysed during the current study available from the corresponding author on reasonable request.
